# Prevalence and risk factors of gestational diabetes mellitus in Nanning, Guangxi, China: a cross-sectional study

**DOI:** 10.3389/fendo.2025.1712920

**Published:** 2025-11-28

**Authors:** Binlin Chen, Rui Jiang, Xiuhua Pan, Jing Huang

**Affiliations:** 1 Department of Clinical Nutrition, Maternity and Child Health Care of Guangxi Zhuang Autonomous Region, Nanning, Guangxi, China; 2 Department of Obstetrics, Maternity and Child Health Care of Guangxi Zhuang Autonomous Region, Nanning, Guangxi, China

**Keywords:** gestational diabetes mellitus, risk factors, prevalence, maternal age, height, Nanning, China

## Abstract

**Objective:**

This study aimed to investigate the prevalence of gestational diabetes mellitus (GDM), its associated risk factors, and perinatal outcomes among pregnant women in Nanning, the capital city of Guangxi, China.

**Methods:**

A retrospective analysis of a cross-sectional dataset was conducted using data from 2003 pregnant women who underwent prenatal care and completed a 75g oral glucose tolerance test (OGTT) at 24–28 weeks of gestation during the year 2021 at the Maternity and Child Health Care of Guangxi Zhuang Autonomous Region in Nanning. Demographic, anthropometric, and clinical data were collected. Statistical analyses included univariate analysis, multivariable logistic regression (adjusted for pre-pregnancy BMI, parity, educational level, and family history of diabetes), and restricted cubic spline (RCS) models to explore dose-response relationships.

**Results:**

A total of 375 cases of GDM were identified, yielding a prevalence of 18.72% (95% CI: 17.10%–20.34%). Women with GDM were significantly older (mean age: 31.62 vs. 30.62 years, *P* < 0.001) and shorter (mean height: 158.1 cm vs. 159.0 cm, *P* < 0.001) compared to non-GDM women. Multivariable logistic regression showed that age ≥ 30 years (OR = 1.61, 95% CI 1.25–2.08, *P* < 0.001) and height < 159.0 cm (OR = 1.27, 95% CI 1.01–1.61, *P* = 0.048) were independent risk factors for GDM. Restricted cubic spline models confirmed a positive dose-response between age and GDM risk, and a linear inverse relationship between height and GDM risk (both *P* for overall < 0.05). Additionally, the GDM group exhibited a higher incidence of preterm birth (8.76% vs. 4.92%, *P* = 0.030).

**Conclusions:**

The prevalence of GDM in Nanning, Guangxi, southern China is high. Older maternal age (≥30 years) and shorter stature (<159.0 cm) are independently associated with increased GDM risk in this urban population. These findings highlight the need for targeted screening and preventive strategies for high-risk groups in Nanning.

## 1 Introduction

Gestational diabetes mellitus (GDM), defined as glucose intolerance with onset or first recognition during pregnancy, represents one of the most common metabolic disorders of pregnancy. It poses substantial risks to both maternal and neonatal health, including preeclampsia, cesarean delivery, macrosomia, neonatal hypoglycemia, and long-term metabolic sequelae for both mother and offspring (1). The global prevalence of GDM has been rising in parallel with increasing rates of obesity, sedentary lifestyles, and advancing maternal age, making it a pressing public health issue worldwide (2).

In China, the rapid socioeconomic development, nutritional transition, and implementation of the universal two-child policy have contributed to a significant increase in the incidence of GDM over recent decades (3). A recent meta-analysis reported a pooled prevalence of 14.8% (95% CI: 12.8%–16.7%) for GDM in mainland China, with individual studies showing rates ranging from 10% to over 24% and marked regional variation (4). For instance, higher rates are often observed in urban areas such as Beijing, while lower rates are reported in less developed and rural regions (5). This geographic heterogeneity reflects differences in lifestyle, dietary patterns, socioeconomic status, and possibly genetic background, underscoring the need for region-specific epidemiological studies.

Nanning, the capital of Guangxi Zhuang Autonomous Region in southern China, is a rapidly urbanizing city characterized by rich ethnic diversity, with significant populations of Zhuang, Yao, Miao, and other minority groups alongside the Han majority. This demographic uniqueness, combined with urban dietary habits—often plant-based but increasingly influenced by westernized diets—may lead to distinct GDM risk patterns that have not been thoroughly investigated. The Maternity and Child Health Care Hospital of Guangxi Zhuang Autonomous Region, located in Nanning, serves as a major referral center, accounting for approximately 15-20% of all births in the city in recent years. Its patient population is ethnically and socioeconomically diverse, providing an ideal setting for examining city-specific factors associated with GDM. A recent study reported an overall GDM prevalence of 8.4% across Guangxi, which included both urban and rural populations (6). This figure is substantially lower than the rates observed in many Chinese metropolises (7, 8), highlighting a potential urban-rural disparity and underscoring the necessity to investigate GDM epidemiology within the specific urban context of Nanning. To date, few comprehensive studies have assessed the prevalence and risk factors of GDM in this key urban population, limiting the development of targeted screening and prevention strategies.

Beyond established factors like age and obesity, emerging evidence suggests that shorter maternal height may also be associated with increased GDM risk (9, 10). However, the underlying mechanisms remain incompletely understood, and this relationship has been underexplored in the unique multiethnic urban context of Southern China.

This study aimed to fill this research gap by conducting a retrospective analysis of a cross-sectional dataset of pregnant women receiving prenatal care in Nanning. Specifically, we sought to: (1) determine the prevalence of GDM; (2) identify independent maternal risk factors—including demographic, anthropometric, and clinical variables; and (3) assess associated perinatal outcomes. We hypothesized that older maternal age and shorter stature would be independently associated with increased GDM risk in this urban population. By leveraging data from a large, contemporary cohort, this research provides timely evidence to inform clinical practice and public health planning aimed at reducing the burden of GDM in southern Chinese urban centers and similar settings.

## 2 Methods

### 2.1 Study design and participants

This retrospective analysis of a cross-sectional dataset included a random sample of 2003 pregnant women. The participants received prenatal care and completed a 75g OGTT at 24–28 weeks of gestation during 2021 at the study hospital. Inclusion criteria were: (1) women aged 17–45 years residing in Nanning (local residents or those with residence duration >6 months); (2) complete prenatal care and medical records. Exclusion criteria included incomplete data (e.g., missing key variables like OGTT results or height) or severe psychiatric disorders that could impede reliable data collection.

### 2.2 Ethical approval

The study protocol was approved by the Ethics Committee of Maternity and Child Health Care of Guangxi Zhuang Autonomous Region (Approval No. [2023-2]6) and strictly adhered to the ethical principles outlined in the Declaration of Helsinki. Data were collected solely from routine clinical care, and no interventions beyond standard medical practice were implemented. Informed consent was waived by the ethics committee due to the retrospective nature of the study.

### 2.3 Data collection

Demographic and clinical data were retrospectively extracted from the hospital information system (HIS). Variables included ethnicity, height, pre-pregnancy weight, educational level, family history of diabetes in first-degree relatives, gestational age, gravidity, parity, and oral glucose tolerance test (OGTT) values. More specifically, height measurement was performed by trained nursing staff using a standardized stadiometer during the initial antenatal consultation at the hospital. The procedure was conducted with participants barefoot, following established protocols, with measurements recorded to the nearest 0.1 cm precision. Pre-pregnancy body mass index (BMI) was categorized according to the Chinese Expert Consensus on Obesity Prevention: underweight (BMI <18.5 kg/m²), normal weight (BMI 18.5–23.9 kg/m²), overweight (BMI 24–27.9 kg/m²), and obesity (BMI ≥28 kg/m²). The 75g OGTT was performed in the hospital’s laboratory following a standardized protocol, which included verification of the 8-hour fast prior to testing.

Regarding data completeness, the vast majority of variables were directly retrieved from the HIS. For the few participants with sporadic missing data (e.g., on educational level or family history), attempts were made to contact them via telephone to complete the information. The final analytical dataset of 2003 participants represents cases with complete data on all variables of interest for this analysis.

### 2.4 Diagnostic criteria for GDM

According to the *IADPSG* criteria, GDM was diagnosed between 24–28 weeks of gestation using a 75 g 2 h oral glucose tolerance test (OGTT) (8). After an 8-hour fast, venous blood samples were collected at fasting, 1-hour, and 2-hour post-glucose ingestion. GDM was defined if any of the following thresholds were met: fasting blood glucose (FBG) ≥5.1 mmol/L, 1-hour glucose ≥10.0 mmol/L, or 2-hour glucose ≥8.5 mmol/L.

### 2.5 Statistical analysis

Data were analyzed using SPSS 25.0 and R 4.4.1. Normally distributed continuous variables were expressed as mean ± standard deviation (SD), while non-normally distributed variables were reported as median (interquartile range, IQR). Group comparisons utilized Student’s t-test (normal distribution) or Mann-Whitney U test (non-normal distribution). Categorical variables were analyzed using chi-square (χ²) or Fisher’s exact tests. Multivariable logistic regression identified independent risk factors for GDM, with results presented as adjusted odds ratios (ORs) and 95% confidence intervals (CIs). The 95% CI for GDM prevalence in Nanning was calculated using the Wilson method. Variables (age, height, BMI, education, parity and family history of diabetes) were included in the regression model. Restricted cubic spline (RCS) models with four knots were employed to assess dose-response relationships between continuous variables (age, height and BMI) and GDM risk. A two-tailed *P*-value <0.05 was considered statistically significant.

## 3 Results

### 3.1 Baseline characteristics

A total of 2003 pregnant women were included in this study, of whom 375 were diagnosed with GDM, corresponding to a prevalence of 18.72% (95% CI: 17.10%–20.34%) in Nanning. The baseline characteristics of the study participants, stratified by GDM status, are compared in **Table 1**. The GDM group had a significantly higher mean age (31.62 ± 4.63 years vs. 30.62 ± 4.94 years, *P* < 0.001) and a greater proportion of women aged ≥30 years (65.87% vs. 55.34%, *P* < 0.001) compared to the non-GDM group. Additionally, women with GDM were shorter in stature (158.1 ± 4.8 cm vs. 159.0 ± 4.9 cm, *P* < 0.001). Furthermore, a significantly higher proportion of women in the GDM group had a height below 159.0 cm compared to the non-GDM group (54.67% vs. 48.71%, *P* = 0.038). No significant differences were observed in ethnicity, pre-pregnancy BMI categories, education level, family history of diabetes in first-degree relatives, parity, or gravidity between the two groups (all *P* > 0.05).

**Table 1:** Comparison of baseline characteristics between women with and without gestational diabetes mellitus (GDM).

Variables	GDM (n=375)	Non-GDM (n=1628)	*P* value
Ethnicity, n (%)			0.295
Han Minority	204 (54.40) 171 (45.60)	934 (57.37) 694 (42.63)	
Age (years)	31.62 ± 4.63	30.62 ± 4.94	<0.001
Age group, n (%)			<0.001
<30 ≥30	128 (34.13) 247 (65.87)	727 (44.66) 901 (55.34)	
Height (cm)	158.1 ± 4.8	159.0 ± 4.9	<0.001
Height classification, n (%)			0.038
<159.0 cm ≥159.0 cm	205(54.67) 170(45.33)	793(48.71) 835(51.29)	
Pre-pregnancy BMI (kg/m^2^)	21.92 ± 4.06	21.84 ± 3.97	0.702
BMI classification, n (%)			0.490
Underweight Normal weight Overweight Obesity	75 (20.00) 200 (53.33) 65 (17.33) 35(9.33)	339 (20.82) 833 (51.17) 328(20.15) 128(7.86)	
Education, n (%)			0.652
College or above High school or below	267 (71.20) 108 (28.80)	1178 (72.36) 450 (27.64)	
Family history of diabetes in first-degree relatives, n (%)	33 (8.80)	120 (7.37)	0.348
Gravidity	2.0 (2.0, 3.0)	2.0 (1.0, 3.0)	0.147
Parity	1.0 (0.0, 1.0)	0.0 (0.0, 1.0)	0.943

BMI, body mass index.

Continuous variables are presented as the mean ± standard deviation (SD), and categorical variables are presented as participants (percentage). Gravidity and Parity are presented as median (IQR) due to non-normal distribution and were compared using the Mann–Whitney U test.

### 3.2 Multivariable logistic regression analysis

Univariate analysis identified age and height as statistically significant variables associated with GDM risk. In the multivariable logistic regression model adjusted for pre-pregnancy BMI, parity, educational level, and family history of diabetes (**Table 2**), age ≥30 years (OR = 1.61, 95% CI: 1.25–2.08, *P* < 0.001) and height <159.0 cm (OR = 1.27, 95% CI: 1.01–1.61, *P* = 0.048) emerged as independent risk factors for GDM. These results underscore the independent contributions of older maternal age and shorter stature to GDM susceptibility in this population.

**Table 2:** Multivariable logistic regression analysis for GDM risk factors.

Variables	β	S.E	Z	*P*	OR (95%CI)
Height (cm)					
≥159.0					1.00 (Reference)
<159.0	0.24	0.12	1.98	0.048	1.27 (1.01 – 1.61)
Age classification (year)					
<30					1.00 (Reference)
≥30	0.48	0.13	3.66	<0.001	1.61 (1.25 – 2.08)
BMI classification					
Normal weight					1.00 (Reference)
Underweight	-0.03	0.15	-0.21	0.835	0.97 (0.72 – 1.31)
Overweight	-0.21	0.16	-1.31	0.192	0.81 (0.59 –1.11)
Obesity	0.07	0.21	0.32	0.751	1.07 (0.71 – 1.62)
Educational level					
College Or Above					1.00 (Reference)
High School Or Below	0.06	0.13	0.46	0.646	1.06 (0.82 – 1.37)
Parity classification (time)					
0 time					1.00 (Reference)
≥1 time	-0.16	0.13	-1.26	0.207	0.85 (0.66 – 1.09)
Family history of diabetes					
No					1.00 (Reference)
Yes	0.07	0.21	0.33	0.739	1.07 (0.71 – 1.63)

OR, Odds Ratio, CI, Confidence Interval.

### 3.3 Dose-response relationships between age, height, BMI and GDM risk

**Figure 1:**
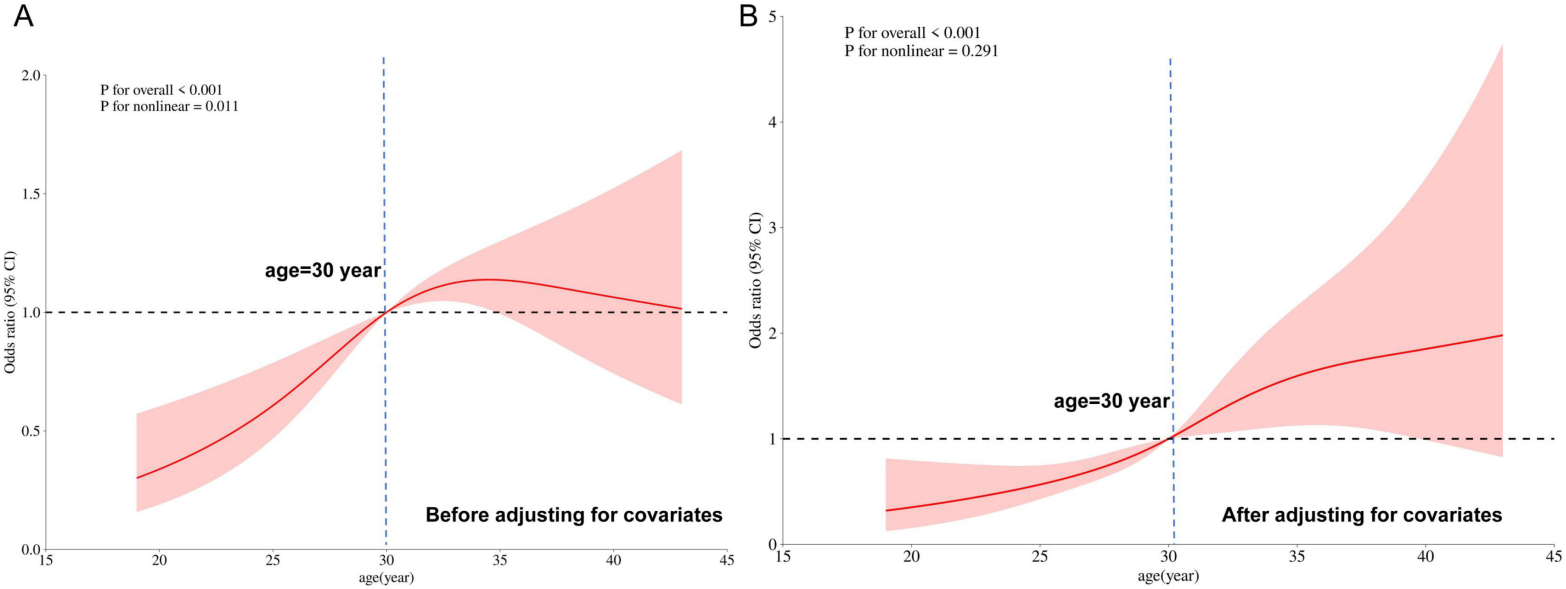
The dose-response relationship between age and risk of GDM. The restricted cubic spline model demonstrates a positive association between maternal age and odds ratio (OR) of GDM. The solid line represents the OR, and the shaded area indicates the 95% confidence interval.

**Figure 2:**
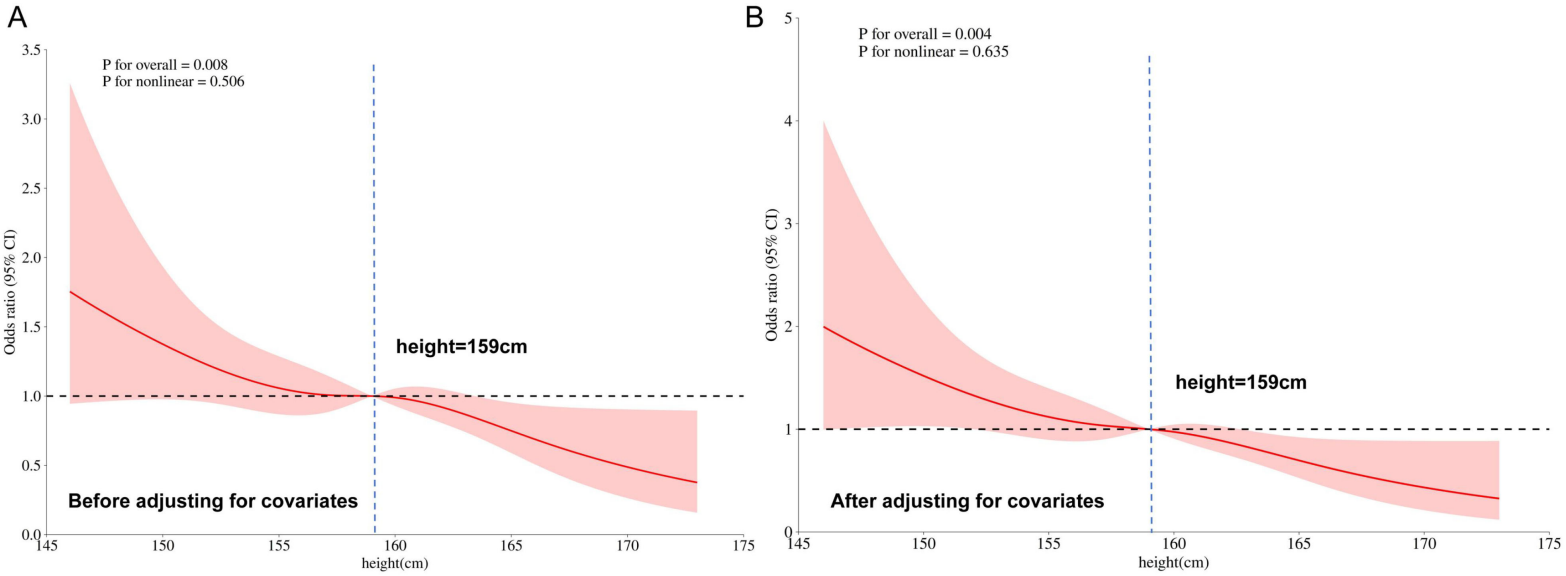
The dose-response relationship between height and the risk of GDM. The restricted cubic spline analysis reveals a linear inverse association between maternal height and OR of GDM. The solid line denotes the OR, with the shaded region representing the 95% confidence interval.

Restricted cubic spline (RCS) models were used to elucidate the dose-response relationships between continuous variables (age, height and BMI) and GDM risk. Analyses were performed both without adjustment and after adjustment for covariates including pre-pregnancy BMI, educational level, gravidity, parity, and family history of diabetes. For age, the RCS model incorporated four knots located at the 5th (23 years), 35th (29 years), 65th (32 years), and 95th (39 years) percentiles. In the unadjusted model, age demonstrated a nonlinear positive association with GDM risk (*P* for overall < 0.001; *P* for nonlinearity = 0.011) (**Figure 1A**). The odds ratio (OR) for GDM was set at 1 at the reference point of 30 years. The risk increased steadily until approximately 35 years of age. After adjusting for covariates, the association between age and GDM risk became approximately linear (*P* for overall < 0.001; *P* for nonlinearity = 0.291), with the reference point remaining at 30 years (OR = 1) (**Figure 1B**). For height, the RCS model also used four knots at the 5th (150 cm), 35th (157 cm), 65th (160 cm), and 95th (167 cm) percentiles. In the unadjusted analysis, height exhibited a linear inverse relationship with GDM risk (*P* for overall = 0.008; *P* for nonlinearity = 0.506), indicating that shorter stature was associated with higher odds of GDM (**Figure 2A**). This linear inverse relationship remained significant after adjustment for covariates (*P* for overall = 0.004; *P* for nonlinearity = 0.635) (**Figure 2B**). In both unadjusted and adjusted models, the OR for GDM was 1 at the reference height of 159.0 cm.

**Figure 3:**
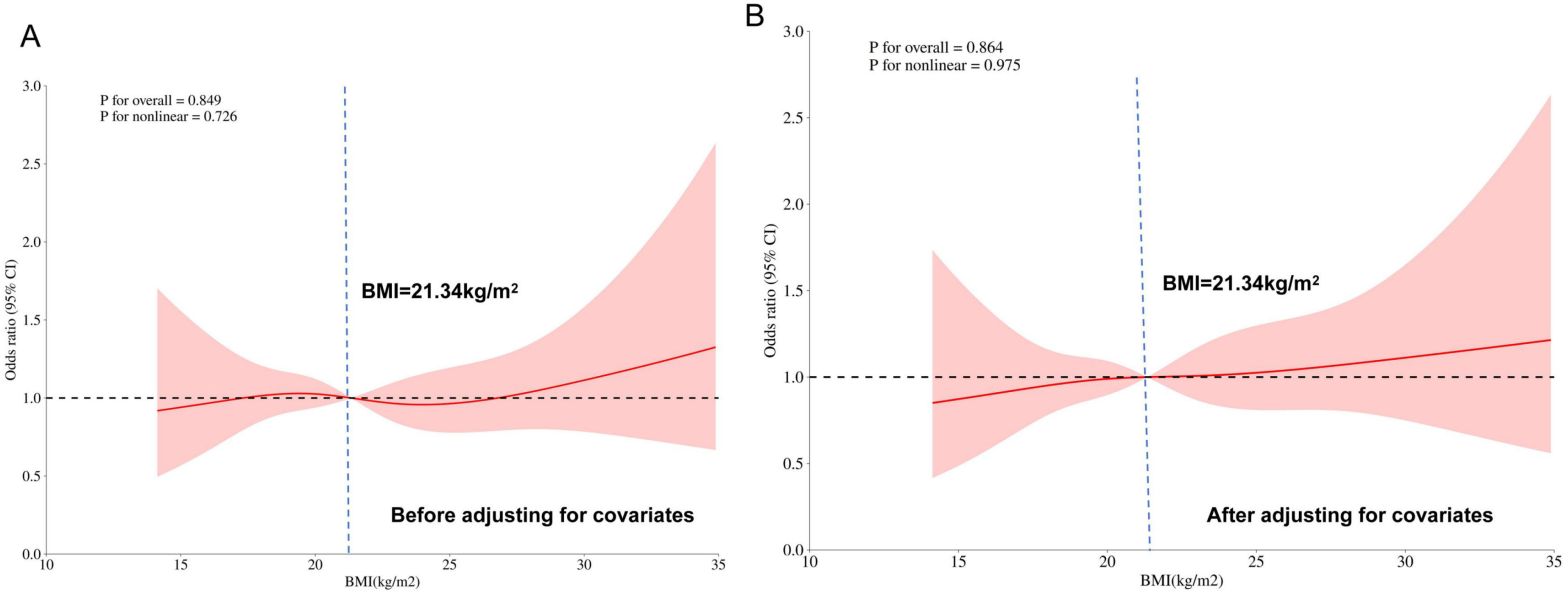
Dose-response relationship between pre-pregnancy BMI and the risk of GDM. Restricted cubic spline models were used to model BMI as a continuous variable. In both panels, the solid line represents the OR, and the shaded area indicates the 95% confidence interval.

For pre-pregnancy BMI, the RCS model incorporated four knots located at the 5th (16.16 kg/m²), 35th (19.88 kg/m²), 65th (23.05 kg/m²), and 95th (29.30 kg/m²) percentiles. The overall BMI distribution of the cohort was relatively low and compact (mean ± SD: 21.85 ± 3.99 kg/m²; median: 21.34 kg/m²; IQR: 19.03 to 24.26 kg/m²). In the unadjusted model, BMI demonstrated no significant association with GDM risk (*P* for overall = 0.849; *P* for nonlinearity = 0.726)(**Figure 3A**). After adjusting for covariates including age, educational level, gravidity, parity, and family history of diabetes, the association remained non-significant (*P* for overall = 0.864; *P* for nonlinearity = 0.975)(**Figure 3B**). These results indicate that, within our study population, pre-pregnancy BMI was not a statistically significant independent predictor of GDM risk when modeled as a continuous variable using a flexible RCS approach.

These findings highlight the differential impacts of age and height on metabolic dysregulation during pregnancy.

### 3.4 Comparison of preterm birth rates between GDM and non-GDM groups

The incidence of preterm birth was significantly higher in the GDM group compared to the non-GDM group (8.76% [33/375] vs. 4.92% [80/1628], *P* = 0.030). This finding underscores the potential adverse perinatal outcomes associated with GDM in this population.

## 4 Discussion

This study revealed a GDM prevalence of 18.72% (95% CI: 17.10%–20.34%) among a sample of pregnant women attending a major referral hospital in Nanning, with older maternal age (≥30 years) and shorter stature (<159.0 cm) identified as independent risk factors. Notably, this prevalence is more than double the 8.4% recently reported for the broader Guangxi region (which included rural areas) (6), highlighting a significant urban-rural disparity and underscoring Nanning’s elevated burden of GDM. These findings align with global trends of rising GDM incidence but also highlight city-specific characteristics that warrant further exploration (11, 12).

The observed prevalence of gestational diabetes mellitus (GDM) (18.72%) aligns with rates reported in Sichuan (18.3%) and Guizhou provinces (18.3%) in prior studies (13, 14), while exceeding the lower prevalence documented in some rural areas (4). This elevated burden in Nanning likely reflects its advanced urbanization, which is characterized not only by lifestyle changes such as sedentary habits and westernized diets but also by a demographic shift toward delayed childbearing. These factors may collectively exacerbate metabolic risks during pregnancy (15). Notably, the association between older maternal age and GDM is consistent with findings from other studies (16). For instance, a recent international study that comprehensively assessed GDM identification and risk factors in a large obstetric cohort also identified older age as a prominent risk factor, reinforcing the consistency of this risk pattern across diverse populations (17). Furthermore, our restricted cubic spline analysis elucidated a dose-response relationship between maternal age and GDM risk. Biologically, aging is linked to progressive declines in β-cell function and insulin sensitivity, compounded by gestational hormonal shifts, which may collectively drive hyperglycemia (18).

The observed inverse association between maternal height and GDM risk in our cohort—with women shorter than 159.0 cm having 27% higher adjusted odds of GDM—is consistent with a growing body of evidence from diverse populations (19–21). While the magnitude of this association is modest, its persistence across studies suggests a potentially meaningful link between shorter stature and increased GDM susceptibility. However, the biological mechanisms underlying this association remain uncertain and largely speculative. Shorter adult height may reflect early-life nutritional limitations or genetic factors that influence both linear growth and glucose metabolism (22), but such pathways could not be directly examined in our retrospective study. Although some studies have hypothesized that shorter individuals may have relatively higher visceral adiposity, which could contribute to insulin resistance (23, 24), this mechanism has not been consistently validated, especially in the context of pregnancy. A meta-analysis found that the inverse relationship between height and diabetes risk was statistically significant only in women (25), but the authors emphasized that sex-specific biological explanations remain hypothetical without direct biomarker or imaging data. To our knowledge, this is one of the few studies to report an independent association between shorter stature and GDM risk in the multiethnic population of Guangxi. Our findings align with those from a large cohort study in Tianjin, China, which identified a similar height threshold (<158.0 cm) as a risk factor for GDM (9). Nonetheless, prospective studies incorporating measures of body composition, early-life exposures, and metabolic biomarkers are needed to clarify the mechanisms behind this association.

Importantly, our study identified a significantly higher incidence of preterm birth in the GDM group compared to the non-GDM group, reinforcing the adverse perinatal consequences of GDM. This finding aligns with global evidence linking GDM to preterm delivery, likely mediated by hyperglycemia-induced inflammation, placental dysfunction, and increased susceptibility to infections (26).

In contrast to many previous reports and meta-analyses that identify pre-pregnancy overweight/obesity (27, 28) and family history of diabetes (29, 30) as risk factors for GDM, our study did not observe significant associations for these variables. The absence of a significant association for pre-pregnancy BMI was further confirmed by RCS analysis treating BMI as a continuous variable. This discrepancy regarding BMI may be attributed to the specific characteristics of our cohort. The BMI distribution was relatively homogeneous and compact (Mean ± SD: 21.85 ± 3.99 kg/m²), with limited representation of individuals in the higher BMI categories, which likely restricted the statistical power to detect its effect. Furthermore, the relationship between BMI and GDM risk might be modulated by other factors not captured in this study, such as specific body composition or dietary patterns. Similarly, the lack of a significant association with a family history of diabetes could be partly related to the overall low reporting rate in our study population and potential under-documentation in retrospective medical records.

The findings from this study carry important implications for public health practice and policy in Nanning and similar urbanizing cities. The high prevalence of GDM (18.72%), significantly exceeding the regional average, underscores the necessity of universal screening using the 75g OGTT as part of routine antenatal care in urban settings. Furthermore, the identification of older maternal age (≥30 years) and shorter stature (<159.0 cm) as independent risk factors suggests that these easily assessable characteristics could be used to stratify pregnant women for more intensive counseling and early preventive measures. Locally, health authorities in Nanning should consider integrating these specific risk factors into city prenatal care guidelines to enhance risk awareness and targeted follow-up. Public health initiatives should also emphasize the importance of pre-pregnancy health education, encourage childbearing at appropriate reproductive ages, and provide personalized management for advanced maternal age pregnancies to mitigate the growing burden of GDM.

This study has several limitations. First, its retrospective and cross-sectional nature precludes inferences about temporal causality and may introduce selection bias. Second, data were restricted to a single tertiary hospital, which may overrepresent urban mothers and limit the generalizability of our findings to rural populations. Third, unmeasured confounders, such as detailed dietary patterns, physical activity, and gestational weight gain, were not analyzed, potentially obscuring additional risk pathways. Future prospective multi-center cohorts should incorporate detailed lifestyle and biochemical measurements.

## 5 Conclusion

In summary, this study conducted in Nanning, the capital city of Guangxi, reveals a high prevalence of GDM (18.72%). Older maternal age (≥30 years) and shorter stature (<159.0 cm) were identified as significant and independent risk factors for GDM in this setting. These findings underscore a substantial urban burden of GDM and highlight the importance of easily assessable maternal characteristics in risk stratification. To effectively address this growing public health concern, targeted screening and tailored preventive strategies should be prioritized for older and shorter pregnant women in urban centers of Southern China. Future research should focus on elucidating the underlying mechanisms, particularly the role of body composition and specific lifestyle factors, and on developing interventions to mitigate GDM risk in these identified high-risk groups.

## Data Availability

The raw data supporting the conclusions of this article will be made available by the authors, without undue reservation.
